# SASdb: a comprehensive database for sex-biased alternative splicing profiles in human tissues

**DOI:** 10.1186/s13293-026-00861-5

**Published:** 2026-02-26

**Authors:** Xi Chen, Yueqi Lu, You Duan, Yongfeng Bai, Weidong Ye, Sijia Chen, Hang Zhou, Heng Xu, Le Xu, Cheng Guo

**Affiliations:** 1 Department of Clinical Laboratory, The Quzhou Affiliated Hospital of Wenzhou Medical University, Quzhou People’s Hospital, Quzhou, 324000 China; 2 Yangtze Delta Region Institute (Quzhou), University of Electronic Science and Technology of China, Quzhou, 324000 China; 3 The Joint Laboratory for Lung Development and Related Diseases of West China Second University Hospital, Sichuan University, School of Life Sciences of Fudan University, West China Institute of Women and Children’s Health, West China Second University Hospital, Sichuan University, Chengdu, 610041 China; 4 College of Agriculture and Biotechnology, Zhejiang University, Hangzhou, 310058 China; 5 Department of Laboratory Medicine, West China Hospital, Sichuan University, Chengdu, 610000 China; 6 Zhejiang Provincial Center for Disease Control and Prevention, Hangzhou, 310058 China

**Keywords:** Alternative splicing, Sex differences, Database, Human tissues, Precision medicine

## Abstract

Alternative splicing (AS) significantly enhances transcriptomic diversity, and its dysregulation is implicated in numerous human diseases. However, no public database systematically compiles sex-related AS events across human tissues. We developed SASdb (http://www.gdbioinfo.top/sasdb), a comprehensive database contains 2,951,059 AS events and 46,418 sex-biased alternative splicing (SAS) events, covering 22 human tissues. SASdb reveals extensive sex-specific splicing patterns, offering new insights into molecular sex differences. A case study on NSCLC-specific SAS events, absent in healthy tissues, highlights their enrichment in cancer-related pathways like autophagy, GPI-anchor biosynthesis, and AMPK/mTOR signaling. SASdb’s intuitive visualization supports research in sex biology and precision medicine.

**Supplementary Information:**

The online version contains supplementary material available at 10.1186/s13293-026-00861-5.

## Background

Alternative splicing (AS) represents one of the most significant post-transcriptional regulatory mechanisms in eukaryotes, enabling a single gene to produce multiple protein isoforms with distinct functions, localization patterns, or regulatory properties [1–3]. This process is governed by cis-regulatory elements and trans-acting splicing factors that drive tissue-specific, developmental, and condition-specific splicing patterns. Although projects like GENCODE and Ensembl annotate multiple isoforms for most human genes, new variants continue to emerge in specific contexts [4–7], indicating substantial undiscovered transcript diversity with potential functional importance.

Beyond generating isoform diversity, aberrant AS is linked to numerous human diseases, including cancer, neurological disorders, muscular dystrophies, and metabolic conditions [8–11]. In cancer, large-scale studies have identified thousands of tumor-associated splicing changes that promote oncogenesis by producing oncogenic isoforms, disrupting tumor suppressors, enhancing metastasis, or conferring therapy resistance [10, 12–16]. This has spurred development of splice-switching oligonucleotides and small-molecule modulators, underscoring the clinical value of comprehensive splicing analyses [15].

Sex differences add further complexity to human biology, influencing disease susceptibility, drug responses, and health outcomes [17–21]. Males and females show distinct patterns in immune function, cardiovascular physiology, neurological development, and metabolism [22, 23], leading to sex-biased disease prevalence (e.g., higher autoimmune rates in females, greater cardiovascular risk in males), differential drug responses, and varying prognoses [24–27]. Recent large-scale transcriptomic studies have begun to uncover the molecular basis of these phenotypic differences, revealing sex-specific gene expression patterns across multiple tissue types [28, 29]. The intersection of alternative splicing and sex biology represents a largely unexplored frontier with significant implications for understanding human health and disease [30, 31].

Despite the importance of AS and sex differences, systematic characterization of sex-biased alternative splicing (SAS) across human tissues is lacking. Existing AS databases, such as MetazExp, MAJIQlopedia, IDeAS, OncoSplicing and ASpedia [13, 16, 32–34], offer valuable general resources but do not focus on sex-biased events. This gap hinders research into sex-specific splicing in physiology, disease, aging, and therapy.

Advances in RNA sequencing (RNA-seq) and tools like rMATS-turbo [35] now enable genome-wide differential splicing analysis across large cohorts. The growing availability of well-annotated public RNA-seq data supports systematic analyses of sex-biased splicing in diverse tissues and conditions.

To address the critical need for systematic SAS resources, we developed SASdb, a comprehensive database dedicated to sex-biased alternative splicing in human tissues. Through rigorous analysis of approximately high-quality RNA-seq samples spanning 22 human tissues, SASdb provides the first systematic catalog of sex-specific splicing events in healthy human tissues. The database integrates advanced visualization tools, functional annotation resources, and statistical analysis capabilities to facilitate exploration of sex-biased splicing patterns and their potential biological significance. To demonstrate the practical utility of SASdb, we present a case study analyzing non-small cell lung cancer (NSCLC) transcriptomes, revealing tumor-specific SAS events and their functional implications for cancer biology.

## Construction and content

### Data collection and preprocessing

We meticulously curated human RNA-seq raw data from the NCBI Gene Expression Omnibus (GEO) and Sequence Read Archive (SRA) databases, selecting samples with detailed metadata on sex, age, tissue type, and health status. To ensure data quality and analytical consistency, we restricted data for each tissue to a single large project to minimize batch effects. Ultimately, we collected 1,044 RNA-seq samples from 22 distinct human tissues, with sex explicitly annotated as male or female (Supplementary Table S1).

Prior to alternative splicing analysis, rigorous quality control and preprocessing of raw RNA-seq data were performed. This process included read quality assessment and filtering using FastQC v0.12.1 (http://www.bioinformatics.babraham.ac.uk/projects/fastqc/), removal of low-quality reads, adapters, and short reads using Trimmomatic v0.39 [36]. High-quality RNA-seq reads were aligned to the reference human genome (GRCh38.p13) using STAR v2.7.9a [37], which effectively handles splice junction alignments. Post-alignment processing included sorting and indexing BAM files using SamTools [38].

### Sex-biased alternative splicing analysis

The core analytical step involved using rMATS [35] to systematically identify sex-biased alternative splicing (SAS) events across tissues. rMATS quantifies five major types of alternative splicing events: Skipped Exon (SE), Retained Intron (RI), Alternative 5’ Splice Site (A5SS), Alternative 3’ Splice Site (A3SS), and Mutually Exclusive Exons (MXE).

For each splicing event, rMATS calculates the Percent Spliced In (PSI) and performs statistical comparisons between conditions (male vs. female), providing P-values and False Discovery Rate (FDR) values. We initially filtered the results using a P-value and FDR threshold of less than 0.05 and displayed all metrics of the filtered results in the database. Researchers can further filter the results based on different metrics (such as P-value, FDR, and PSI difference).

### Database architecture and implementation

SASdb is an open-access database built using open-source softwares. The database runs on a CentOS server, based on the Django (3.0.5) web framework, developed using Python (3.8) programming language. All integrated datasets are imported into a MySQL (5.6.49) relational database, with text search functionality based on Django’s ORM model through in-house Python scripts implementing gene search and tissue search functions.

The web interface is developed based on the Bootstrap (3.3.7) framework, providing dynamic page layouts based on user monitor resolution. Frontend visualization is implemented using HTML + CSS + JavaScript, generating interactive dynamic graphics through Highcharts (10.0.0), ECharts (6.0.0), amCharts (5.13.4), and Plotly (v3.0.1) packages.

The database integrates multiple online analysis functions for functional enrichment and clustering analysis. The former includes KEGG (Kyoto Encyclopedia of Genes and Genomes) and GO (Gene Ontology) functional annotation and enrichment analysis based on the clusterProfiler (4.6.2) [39] package. The latter includes clustering methods such as PCA, PCoA, t-SNE, UMAP, K-Means, Hierarchical K-Means, and Fuzzy Clustering, implemented using packages including Rtsne (0.16), umap (0.2.10.0), factoextra (1.0.7), cluster (2.1.4), and Mfuzz (2.58.0) [40–43].

### Pipeline validation

To rigorously assess the biological reliability and accuracy of the SASdb pipeline, we performed a comprehensive validation analysis using established high-confidence sex-biased splicing markers.

First, as a positive control for sex-exclusive events, we examined *XIST*, the master regulator of X-chromosome inactivation. Our pipeline successfully recapitulated the expected female-specific pattern: robust junction read counts and near-complete exon inclusion were observed exclusively in female samples, whereas male samples showed zero signal across all relevant junctions (Supplementary Figure S1). This binary presence/absence pattern validates the accuracy of our sample sex annotations.

Second, to demonstrate the pipeline’s sensitivity in detecting quantitative splicing shifts on autosomes, we analyzed *BMPR1B* (*Bone Morphogenetic Protein Receptor Type 1B*) on Chromosome 4, a gene implicated in reproductive hormone signaling. SASdb identified a dramatic isoform switch: females predominantly exhibited the constitutive exon inclusion form (Median PSI > 0.9), while males favored the exon-skipped isoform with significantly lower inclusion levels (Median PSI < 0.4; |ΔPSI| > 0.5, FDR < 0.001) (Supplementary Figure S2). This clear, statistically robust event demonstrates the capability of our workflow to quantify biological sex differences beyond sex chromosomes.

Furthermore, broader validation confirmed that the database successfully captures other well-characterized sex-biased splicing genes, such as the X-chromosome escapee *KDM6A* and the synaptic adhesion molecule *NRXN3*. These findings collectively provide strong evidence for the biological relevance and algorithmic precision of the sex-biased splicing events cataloged in SASdb.

## Utility and discussion

### Database features and user interface

**Fig. 1:**
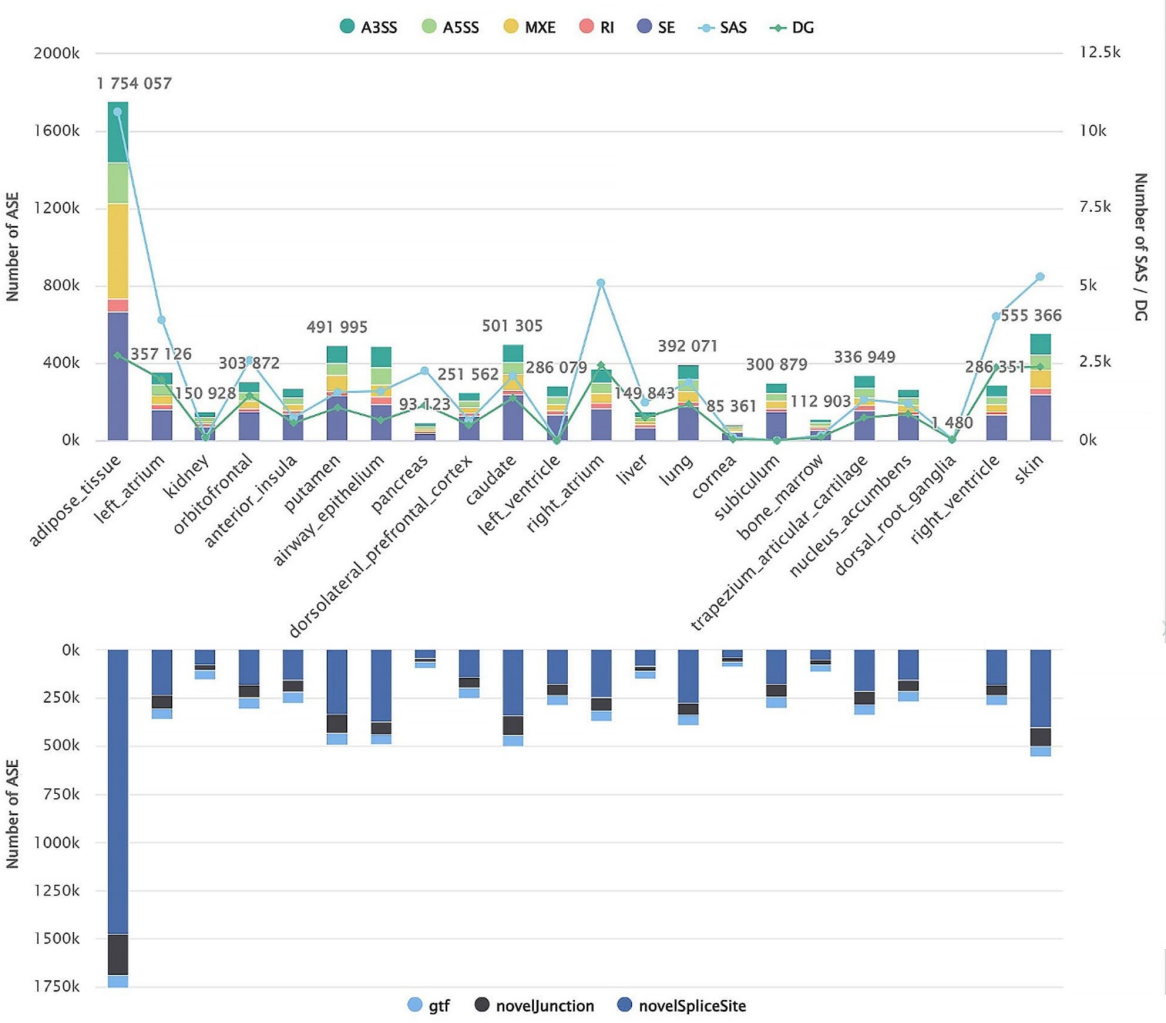
Overview of SASdb. The columns in the figure show the number of ASEs in each type of tissue. The colors in the upper part represent the ASE types, while the colors in the lower part represent the sources of the ASEs. The lines in the figure show the number of SASs and DGs (Differential Genes) in each type of tissue

SASdb catalogs 46,418 sex-biased alternative splicing (SAS) events from 2,951,059 alternative splicing (AS) events across 22 human tissues (Fig. 1). It offers robust visualization and analytical tools, enabling comprehensive exploration of sex-specific splicing patterns to advance research in molecular sex differences and precision medicine.

**Fig. 2:**
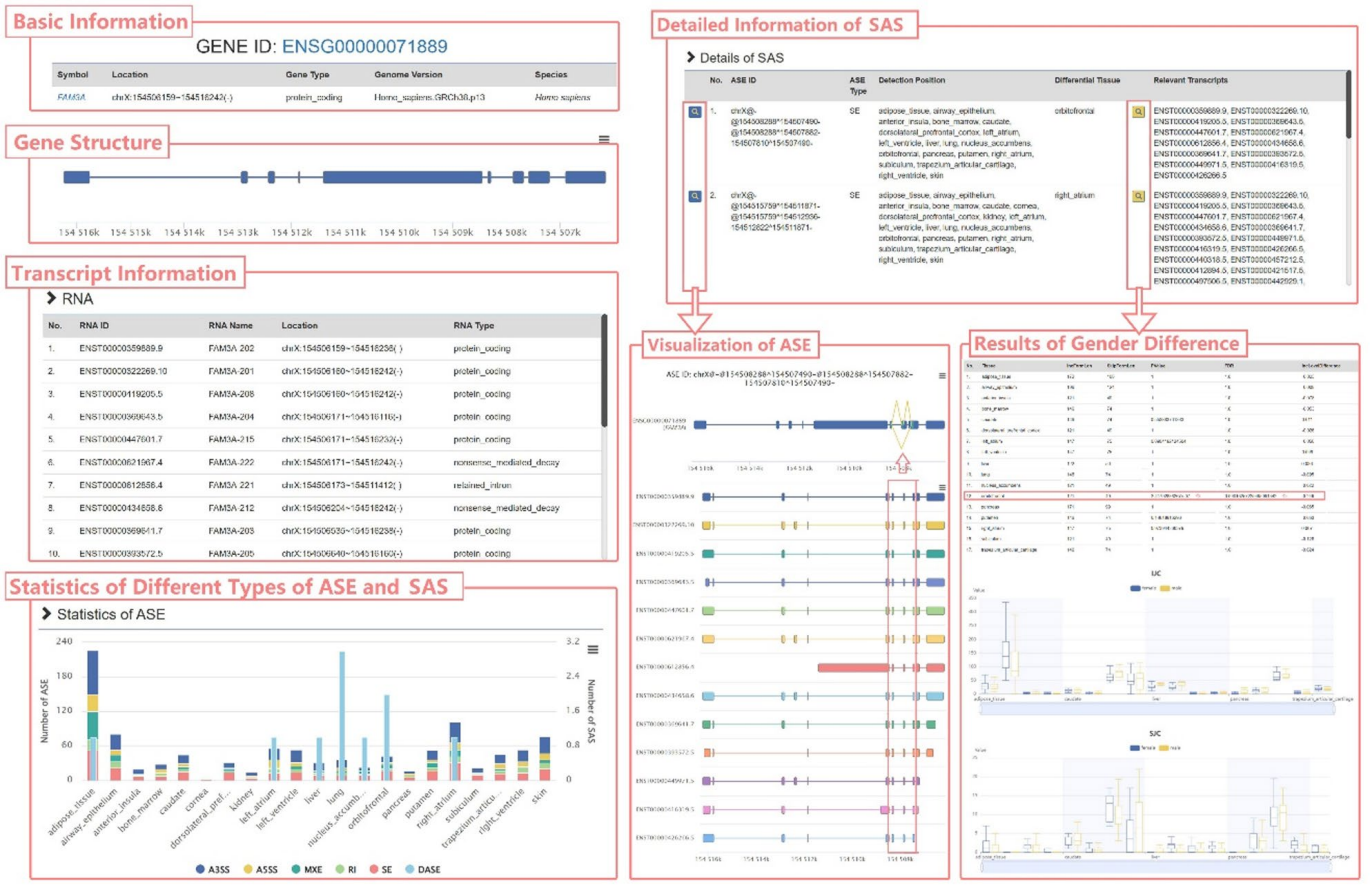
The detail page of gene FAM3A. The page content covers multiple information and visualization modules, including basic information of *FAM3A*, gene structure visualization, transcript information, statistics of ASE and SAS, and detailed information of SAS. Users can click the buttons in the table to browse the dynamic pictures of ASE visualization and its performance in sex difference analysis

The gene detail page, exemplified by *FAM3A* (an X-linked gene encoding a cytokine-like protein with established roles in metabolic regulation, including glucose homeostasis and insulin signaling), displays basic gene information including symbol, location, and gene type, with gene structure visualization (Fig. 2). Users can browse all transcript information related to *FAM3A* through a table interface. A dual-coordinate bar chart shows the statistical distribution of different types of ASE detected in various tissues for *FAM3A*, including the number of SAS statistics.

For detailed SAS investigation, users can browse detailed information for each SAS on the page, including related visualization results and sex difference analysis results. For example, in an SE-type ASE detected in *FAM3A* (ASE ID: chrX@-@154508288^154507490-@154508288^154507882 − 154507810^154507490-), which contains 13 transcripts with two splicing results, this ASE shows significant sex differences in the putamen (Fig. 2).

**Fig. 3:**
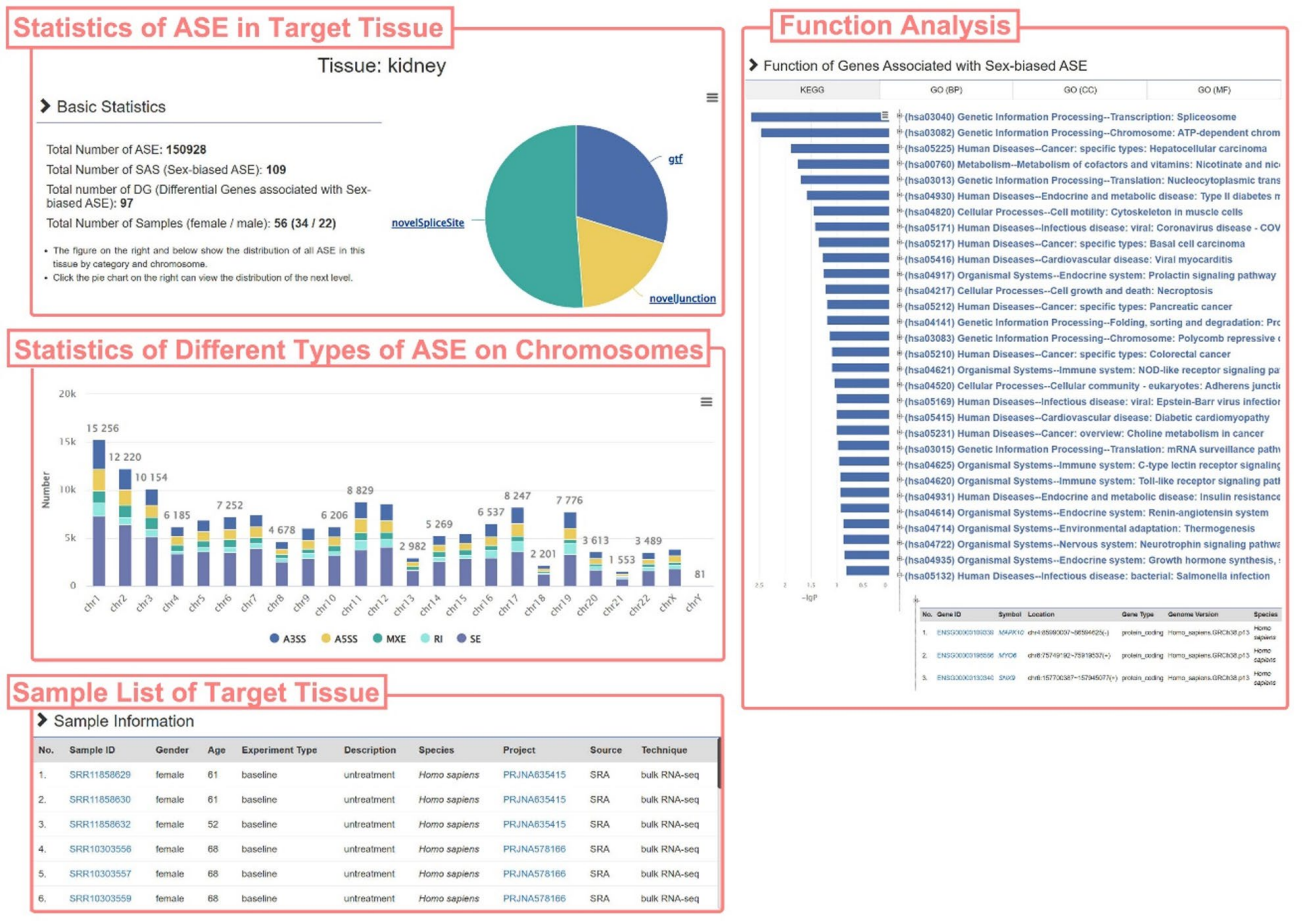
The detail page of kidney. The page content includes the statistics of ASE detected in this tissue, sample information, and the results of function analysis based on DGs

The tissue detail page, exemplified by kidney, allows users to first browse basic statistical information, including the number of ASEs detected in kidney tissue (150,928), the number of SASs (109), and the number of related differential genes (97). The page displays the distribution of five types of ASEs detected in kidney tissue across chromosomes through bar charts. Users can browse all kidney tissue sample information in SASdb through the sample list table (Fig. 3).

For further exploration of kidney-related sex-biased ASE molecular functions, the page displays results of KEGG and GO enrichment analyses based on 97 differential genes. Users can browse information on all significantly enriched pathways and terms, and expand related gene lists by clicking on the nodes of the collapsible tree.

### Methodological considerations for alternative splicing detection

SASdb relies exclusively on rMATS-turbo for the detection and quantification of alternative splicing events. This decision was driven by several key advantages that make rMATS particularly suitable for large-scale, reproducible sex-biased splicing analysis across thousands of samples. rMATS is a mature, widely adopted tool with extensive validation in large cohorts such as TCGA and GTEx, offering robust statistical modeling of junction reads for the five classical splicing event types (SE, MXE, A5SS, A3SS, RI). Its computational efficiency enabled efficient processing of our dataset, and its support for novel junction detection (via the --novelSS option, which was enabled in our pipeline) allowed identification of previously unreported sex-biased events. Additionally, using a single, uniform algorithm ensures consistency in event definition, PSI calculation, and significance testing across all tissues, facilitating direct comparability— a critical feature for a reference database.

### Case study: NSCLC-Specific sex-biased alternative splicing

To demonstrate the practical utility of SASdb, we conducted a comprehensive case study analyzing non-small cell lung cancer (NSCLC) transcriptomes. We downloaded male and female NSCLC transcriptome data (Supplementary Table S2) and applied the same methodology to analyze sex-biased alternative splicing. Through the joint analysis module in the analysis section of the database, we identified NSCLC-specific sex-biased alternative splicing genes (those showing sex-biased alternative splicing in NSCLC but not detected in healthy tissues) and performed functional analysis using the gene function analysis module in the analysis section of the database.

#### NSCLC-specific SAS events and functional enrichment

Using rMATS with the filtering criteria of p-value < 0.05, FDR < 0.01, and absolute inclusion difference (abs(inc_diff)) > 0.1, we identified five types of NSCLC-specific sex-biased alternative splicing events, including 31 Alternative 3’ Splice Site (A3SS), 48 Alternative 5’ Splice Site (A5SS), 24 Mutually Exclusive Exons (MXE), 142 Skipped Exon (SE), and 72 Retained Intron (RI). Through the joint analysis module in the analysis section of the database, we further identified NSCLC-specific sex-biased alternative splicing genes (involving 29 A3SS, 47 A5SS, 22 MXE, 69 RI, and 131 SE). These alternative splicing events showed no obvious sex differences in healthy populations, indicating their NSCLC-specific characteristics.

Functional analysis reveal that 31 A3SS events across 29 genes, enriched in pathways like autophagy-animal, GPI-anchor biosynthesis, longevity regulation, nucleocytoplasmic transport, and AMPK/mTOR signaling, with GO terms indicating roles in intracellular transport, Golgi localization, glycolipid metabolism, lamin binding, and transmembrane transporter activity; 48 A5SS events in 47 genes, associated with COVID-19, Herpes simplex virus 1 infection, ubiquitin-mediated proteolysis, and HIF-1 signaling, alongside cytosolic large ribosomal subunit and clathrin-coated endocytic vesicle components; 24 MXE events in 22 genes, linked to porphyrin metabolism and ubiquitin-mediated proteolysis, with functions in calcium-gated channel and ATPase activator activities; 142 SE events affecting 131 genes, enriched in transport vesicle and synaptic vesicle membranes; and 72 RI events in 69 genes, tied to thyroid hormone synthesis, apoptosis, RNA polymerase complex, and TFIIH holo complex, highlighting their NSCLC-specific and sex-biased nature (Supplementary Figure S3-7).

The discovery of NSCLC-specific sex-biased alternative splicing events reveals critical molecular distinctions in how NSCLC manifests in males versus females, impacting pathways such as metabolism, immune response, hypoxia adaptation, protein degradation, ion channel function, vesicular transport, hormonal signaling, and transcriptional regulation. Clinically, these insights could drive the development of sex-specific diagnostic biomarkers or targeted therapies, such as inhibitors of autophagy or HIF-1 signaling, to address disparities in NSCLC outcomes. Biologically, these findings emphasize alternative splicing’s role in tumor heterogeneity, potentially explaining sex-based differences in NSCLC progression and incidence, and opening avenues for investigating hormonal or epigenetic influences on splicing regulation in cancer. These NSCLC-specific events highlight the value of using healthy tissue baselines in SASdb to identify disease-associated sex differences that would otherwise remain undetected.

### Database impact, limitations and future directions

The construction of SASdb fills a critical gap in sex biology research, providing researchers with unprecedented access to systematic sex-biased alternative splicing data. The database’s user-friendly interface and comprehensive analysis tools facilitate exploration of sex-specific molecular mechanisms across multiple tissues and disease contexts.

The NSCLC case study demonstrates the database’s potential for identifying disease-specific sex differences that may inform precision medicine approaches. The tumor-specificity of these alternative splicing events supports their potential as sex-biased biomarkers for NSCLC.

While SASdb provides the first comprehensive resource for sex-biased alternative splicing across human tissues, several limitations should be noted. First, our analyses rely on publicly available RNA-seq data from GEO/SRA, where metadata (e.g., age, RNA integrity number [RIN], post-mortem interval, ancestry) are often incomplete or heterogeneous across studies. This precluded uniform covariate adjustment for potential confounders known to influence transcriptomic profiles, such as age, batch effects, or health status. Although we mitigated technical batch effects by restricting each tissue to a single large project—prioritizing internal consistency and minimizing noise from multi-center data—this strategy inevitably imposes a trade-off. It may reduce sample diversity, limit generalizability, and potentially conflate project-specific technical factors with biological signals in inter-tissue comparisons. Therefore, we advise users to exercise caution when interpreting results, particularly regarding cross-tissue comparisons, as observed differences may partially reflect project-specific biases rather than purely biological distinctions.

Second, sex-balanced sampling was not always achievable within individual projects, and subtle confounding by unrecorded variables cannot be fully excluded. Users are therefore advised to consider biological covariates (e.g., age, hormonal status) when interpreting or validating individual sex-biased events, particularly in downstream experimental studies.

Finally, regarding the methodology, reliance on a single splicing detection algorithm (rMATS) represents a limitation in the current implementation. Although we utilized the --novelSS option to improve detection, certain classes of splicing variation—such as highly local, complex, or annotation-independent events—may still be underrepresented. Consequently, the current catalog may not capture the complete landscape of sex-biased splicing. Ongoing and future expansions will address these limitations through multi-tool integration and incorporation of additional datasets.

Future developments will include continuous updates with newly released RNA-seq datasets, integration of multi-tool splicing analyses for enhanced event coverage, incorporation of additional multi-omics layers (e.g., splicing factor expression, genetic regulation), and expansion to more disease contexts beyond NSCLC.

## Conclusions

Through systematic analysis of large-scale RNA-seq data from 22 human tissues, we have constructed SASdb, the first comprehensive database dedicated to sex-biased alternative splicing in human tissues. This resource reveals widespread sex-specific splicing patterns and provides novel insights into molecular differences between sexes in both physiological and pathological states.

The practical utility of SASdb is demonstrated through our NSCLC case study, which identified cancer-specific sex-biased alternative splicing events enriched in key cancer pathways. These findings highlight the potential of alternative splicing as a molecular basis for sex differences in disease susceptibility and treatment response.

SASdb serves as a valuable public resource that will accelerate research in sex biology, facilitate the development of sex-specific biomarkers, and support the advancement of precision medicine strategies. The database’s comprehensive coverage, user-friendly interface, and integrated analysis tools make it an essential resource for understanding the molecular basis of sex differences in human health and disease.

## Supplementary Information

Below is the link to the electronic supplementary material.

Supplementary Material 1.

Supplementary Material 2.

Supplementary Material 3.

Supplementary Material 4.

Supplementary Material 5.

## Data Availability

The datasets generated and analysed during the current study are available in the SASdb ( http://www.gdbioinfo.top/sasdb ).

## Abbreviations

AS: Alternative splicing
SAS: Sex-biased alternative splicing
DGs: Differential genes
RNA-seq: RNA sequencing
NCBI: National center for biotechnology information
GEO: Gene expression omnibus
SRA: Sequence read archive
rMATS: Replicate multivariate analysis of transcript splicing
SE: Skipped exon
RI: Retained intron
A5SS: Alternative 5’ splice Site
A3SS: Alternative 3’ splice Site
MXE: Mutually exclusive exons
PSI: Percent spliced in
FDR: False discovery rate
GO: Gene ontology
KEGG: Kyoto encyclopedia of genes and genomes
BP: Biological process
CC: Cellular component
MF: Molecular function
NSCLC: Non-small cell lung cancer
