# Supplementary material for: SASdb: a comprehensive database for sex-biased alternative splicing profiles in human tissues
Source: Biol Sex Differ. 2026 Feb 26;17:60. doi: 10.1186/s13293-026-00861-5 (PMC13041291; doi:10.1186/s13293-026-00861-5)
Supplement: Supplementary file 3 — Supplementary Material 3. [file 13293_2026_861_MOESM3_ESM.docx]

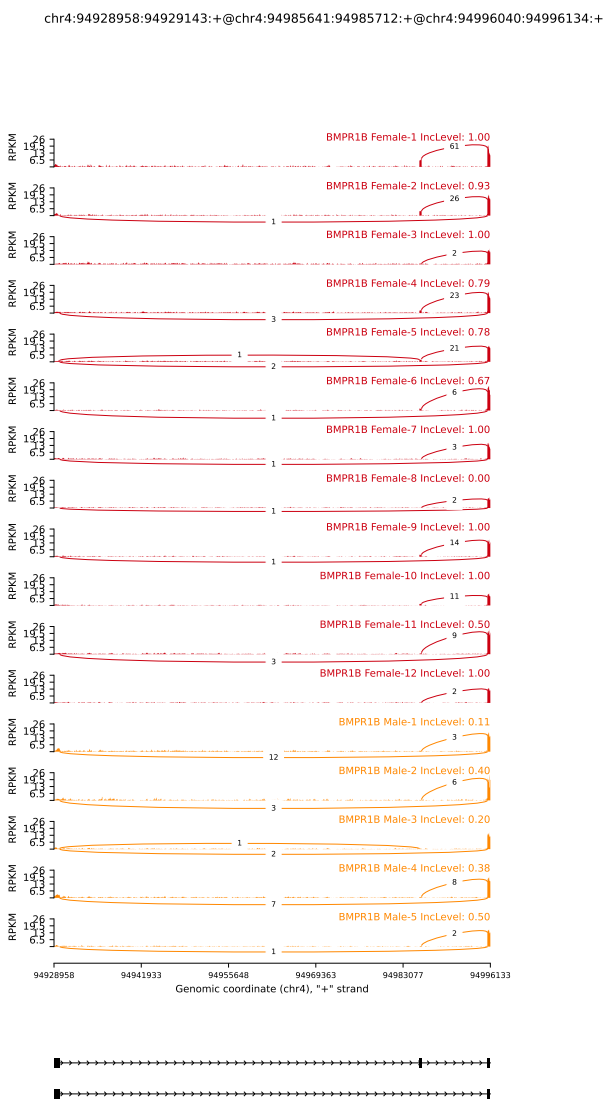


**Figure S2. Sashimi plot of the *BMPR1B* gene.**

Female samples (Red) predominantly show high inclusion levels of the alternative exon, whereas male samples (Orange) show a marked reduction in inclusion (skipping isoform). Numbers on arcs indicate junction read counts; numbers next to sample names indicate PSI (Percent Spliced In) values calculated by rMATS.
