# Supplementary material for: SASdb: a comprehensive database for sex-biased alternative splicing profiles in human tissues
Source: Biol Sex Differ. 2026 Feb 26;17:60. doi: 10.1186/s13293-026-00861-5 (PMC13041291; doi:10.1186/s13293-026-00861-5)
Supplement: Supplementary file 4 — Supplementary Material 4. [file 13293_2026_861_MOESM4_ESM.docx]

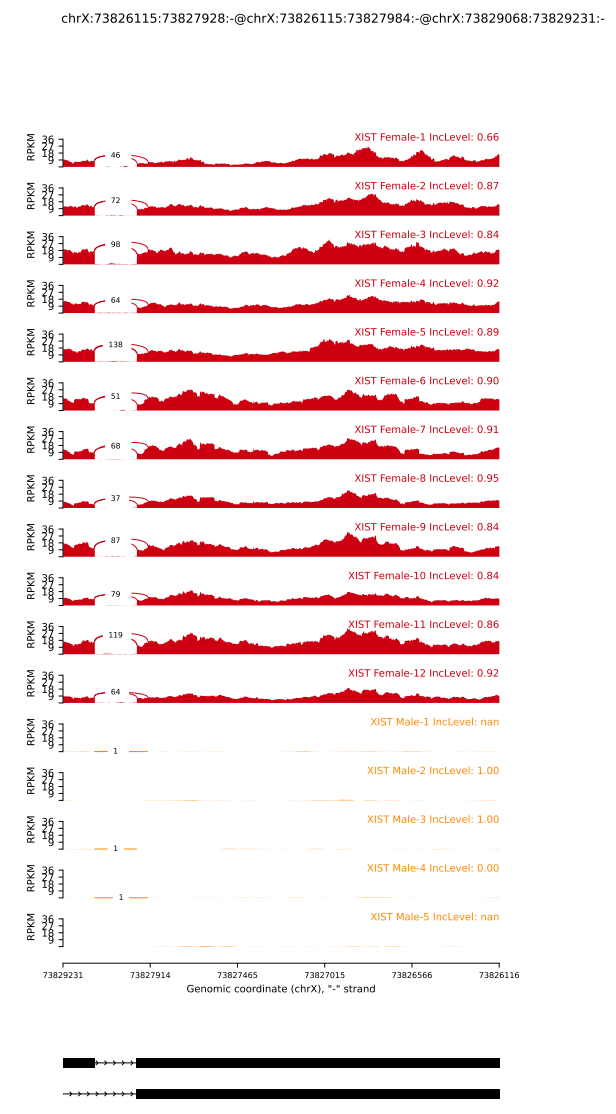


**Figure S1. Sashimi plot of the *XIST* gene.**

Sashimi plot visualization of the *XIST* gene (X chromosome) showing female-specific expression and splicing. Red tracks represent female samples with robust junction coverage (Inclusion Level ~0.8–0.9), while orange tracks represent male samples with negligible read counts, confirming the accuracy of sex metadata. Numbers on arcs indicate junction read counts; numbers next to sample names indicate PSI (Percent Spliced In) values calculated by rMATS.
