# Supplementary material for: SASdb: a comprehensive database for sex-biased alternative splicing profiles in human tissues
Source: Biol Sex Differ. 2026 Feb 26;17:60. doi: 10.1186/s13293-026-00861-5 (PMC13041291; doi:10.1186/s13293-026-00861-5)
Supplement: Supplementary file 5 — Supplementary Material 5. [file 13293_2026_861_MOESM5_ESM.docx]

**Figure S3. The enrichment analysis results of NSCLC-specific sex-biased A3SS related genes**

| **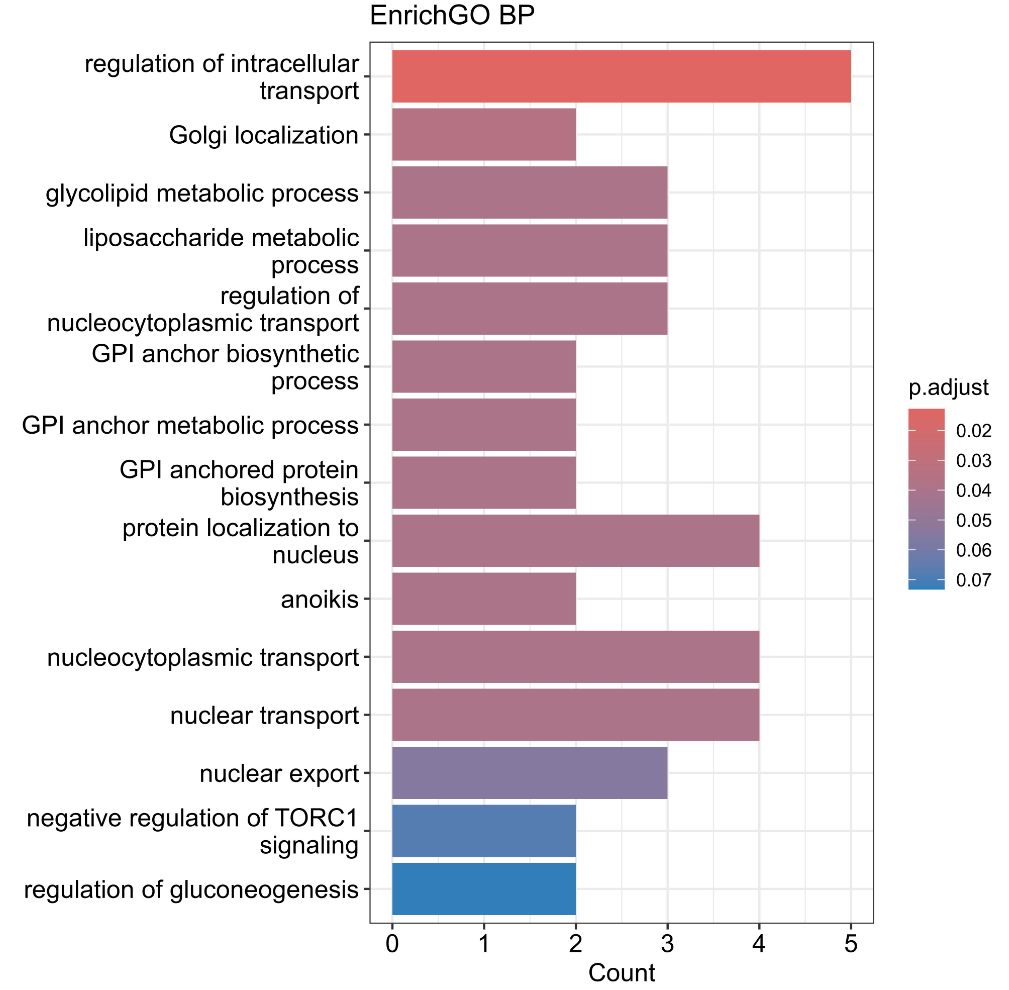** | **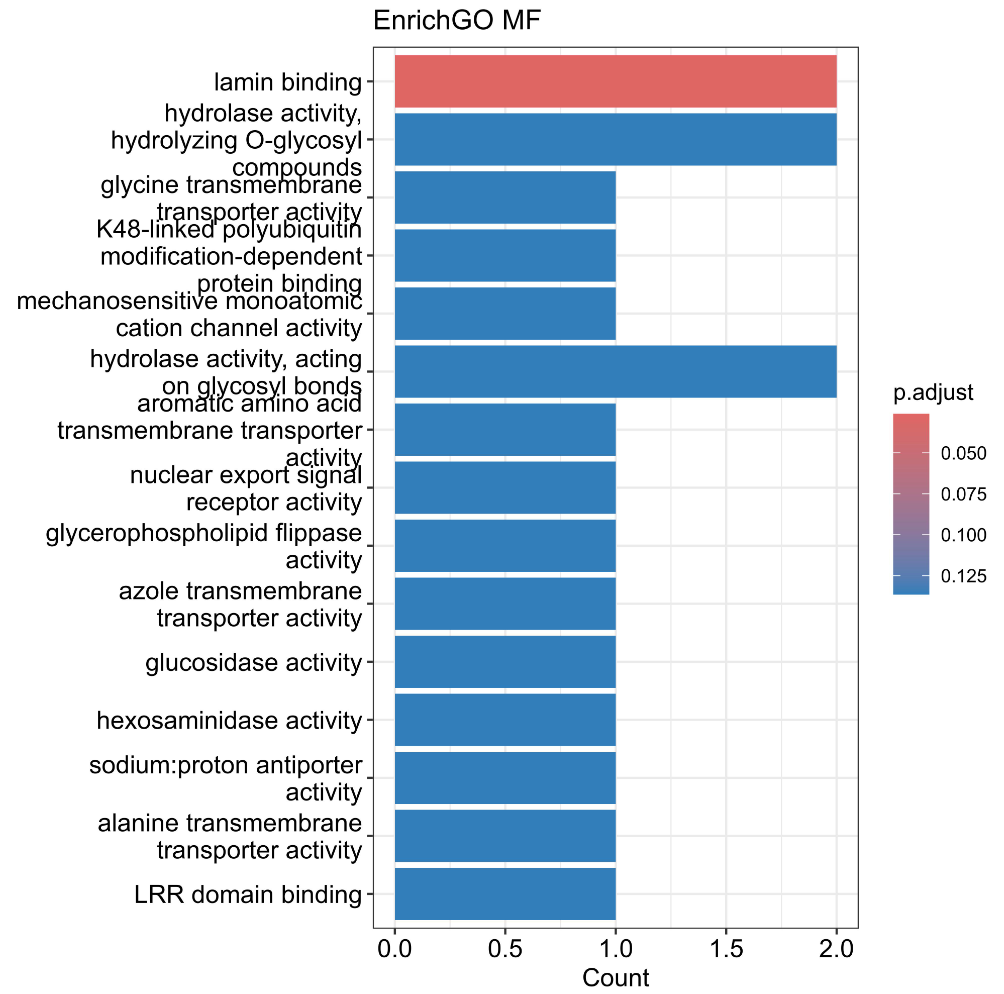** |
| --- | --- |

**
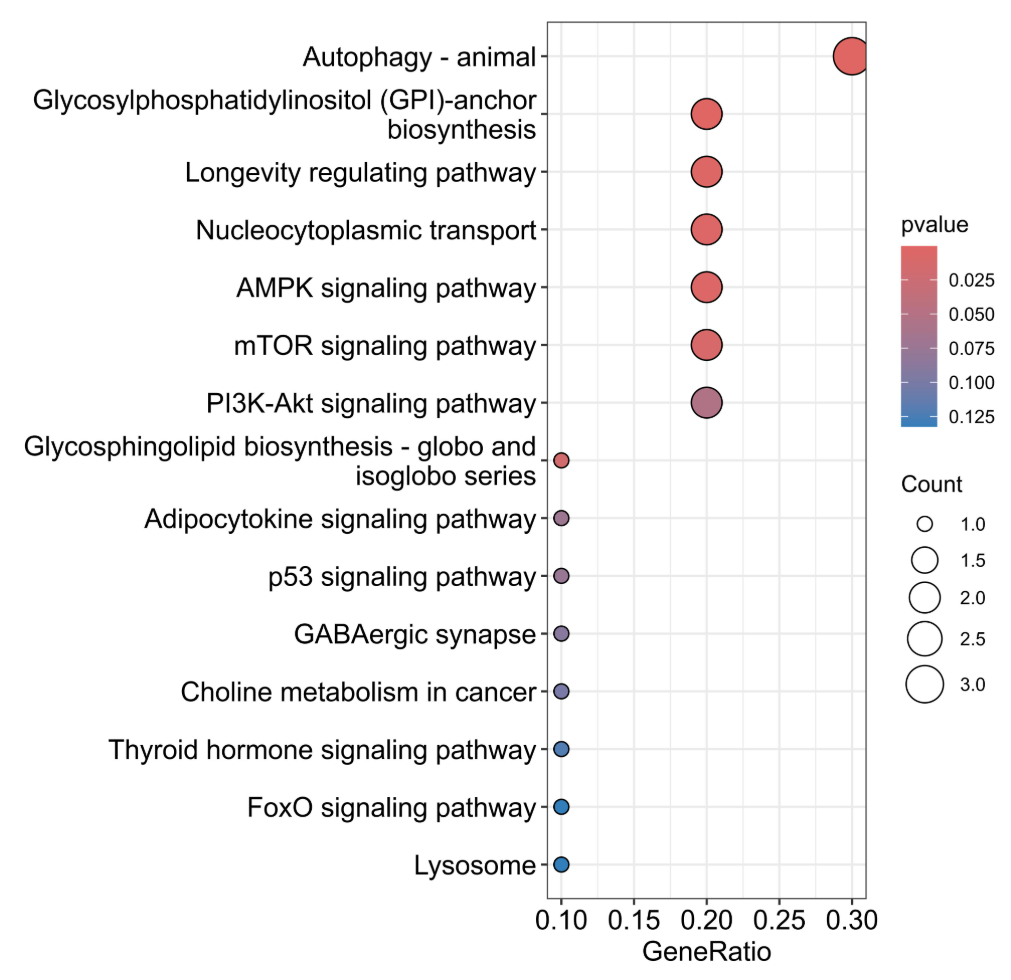
**

**Figure S4. The enrichment analysis results of NSCLC-specific sex-biased A5SS related genes**

| **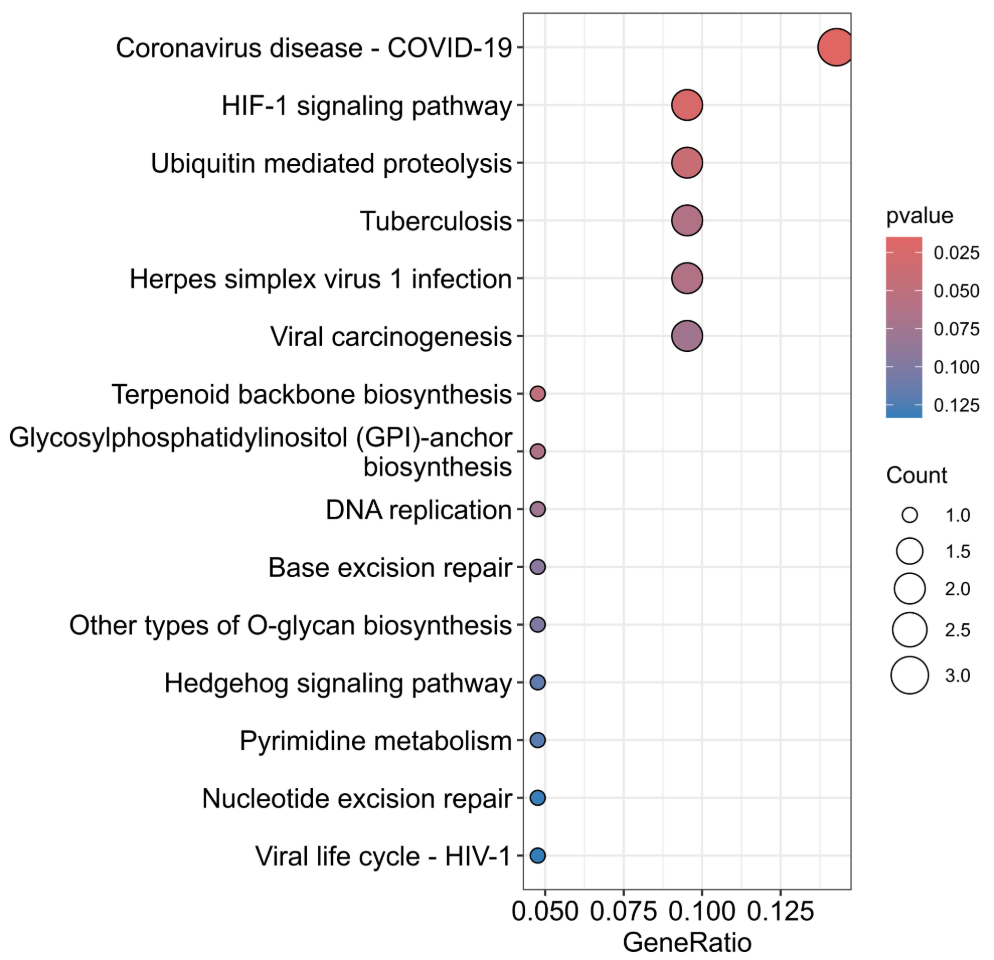** | **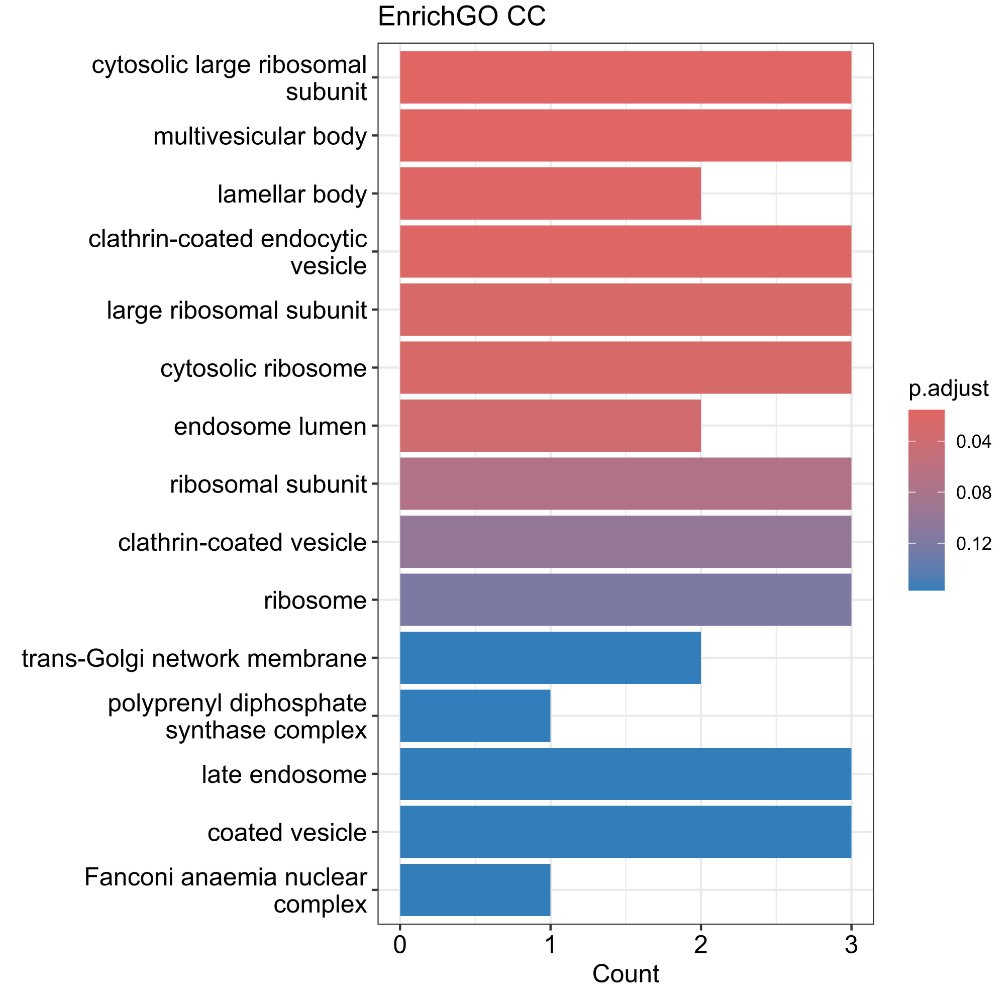** |
| --- | --- |

**Figure S5. The enrichment analysis results of NSCLC-specific sex-biased MXE related genes**

| **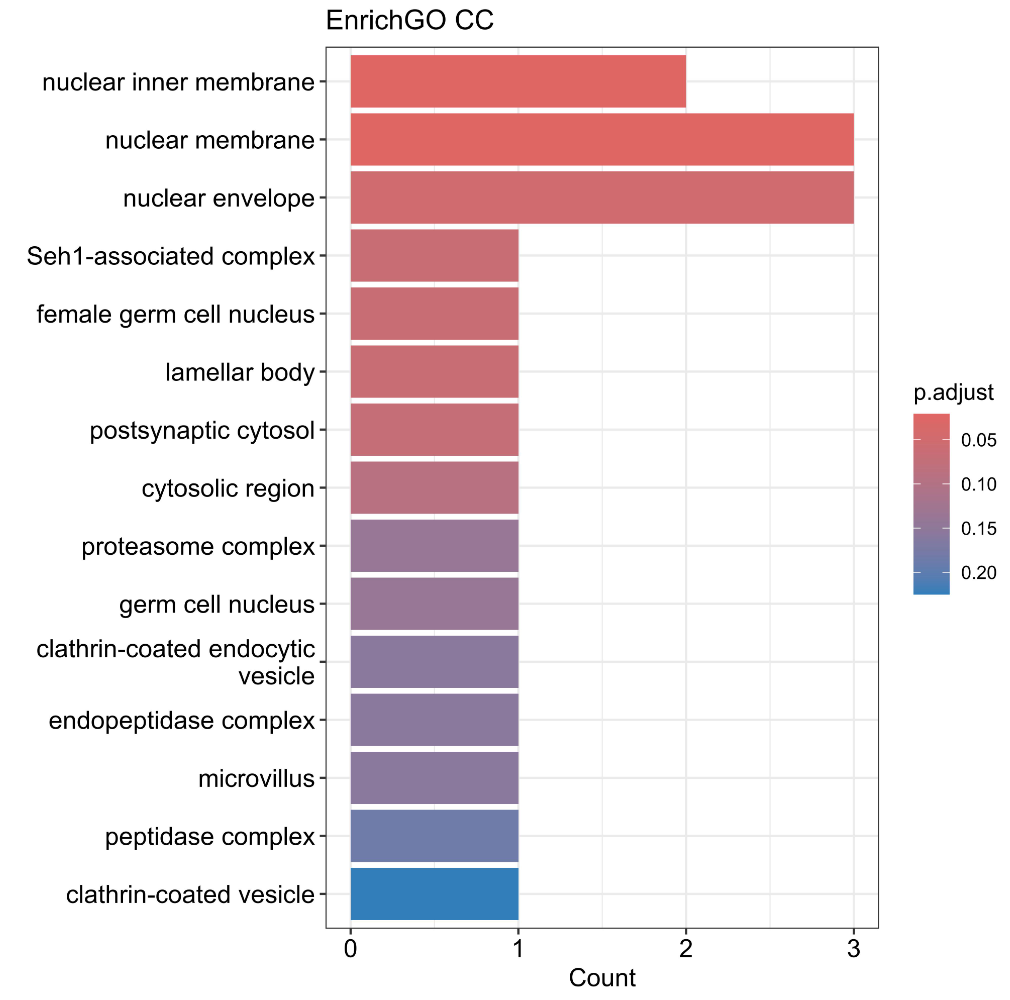** | **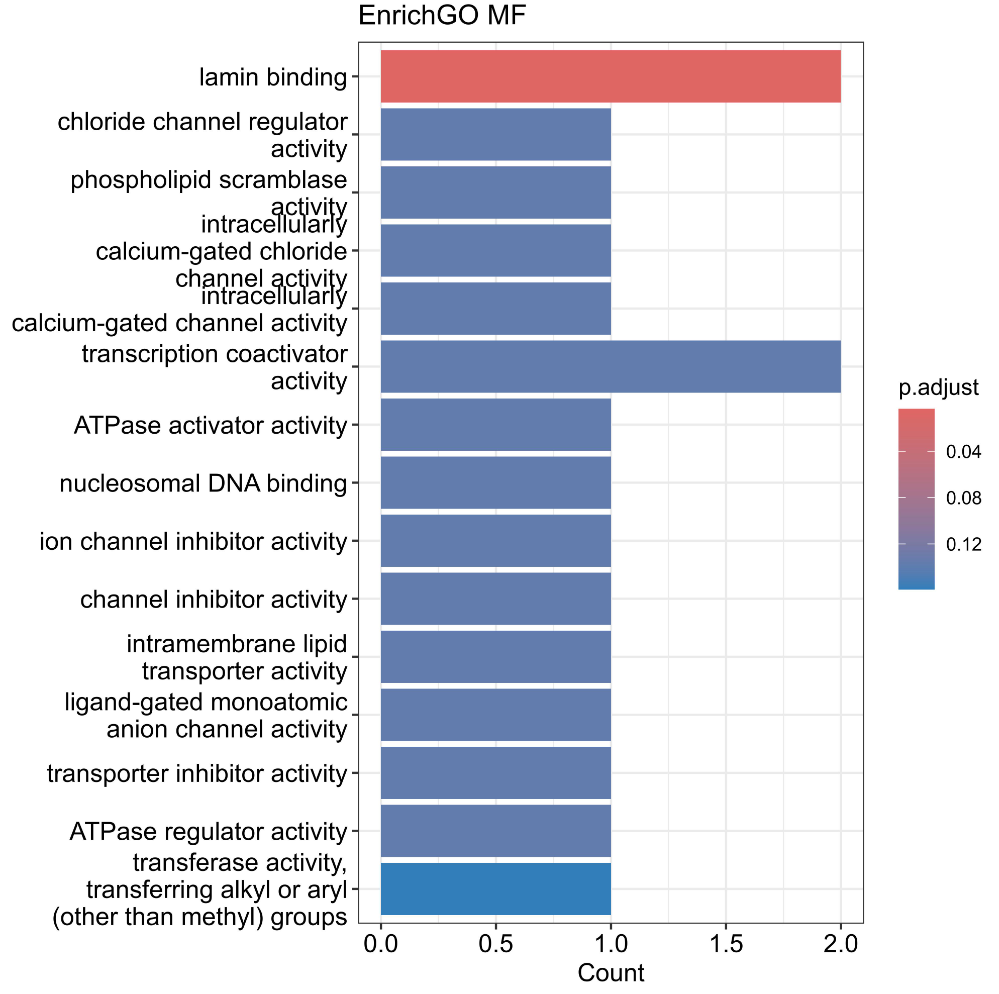** |
| --- | --- |

**
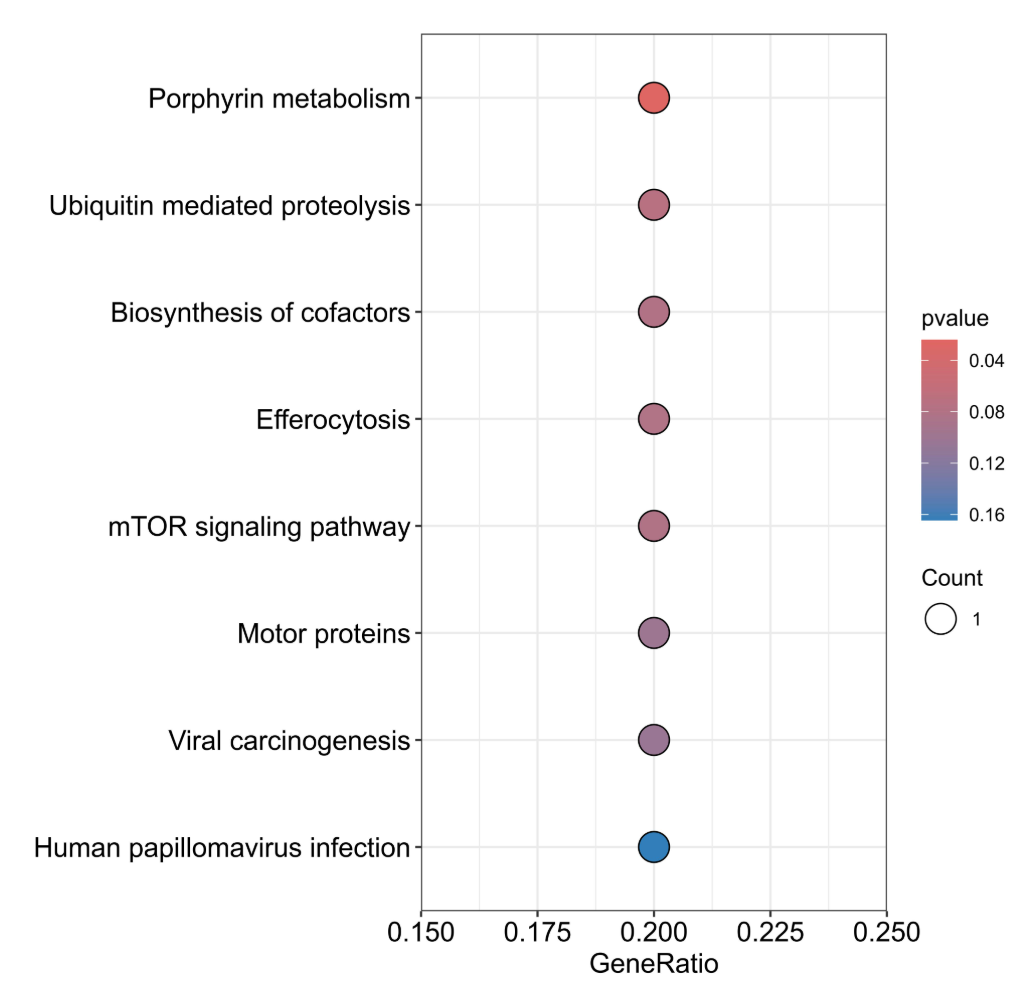
**

**Figure S6. The enrichment analysis results of NSCLC-specific sex-biased SE related genes**

**
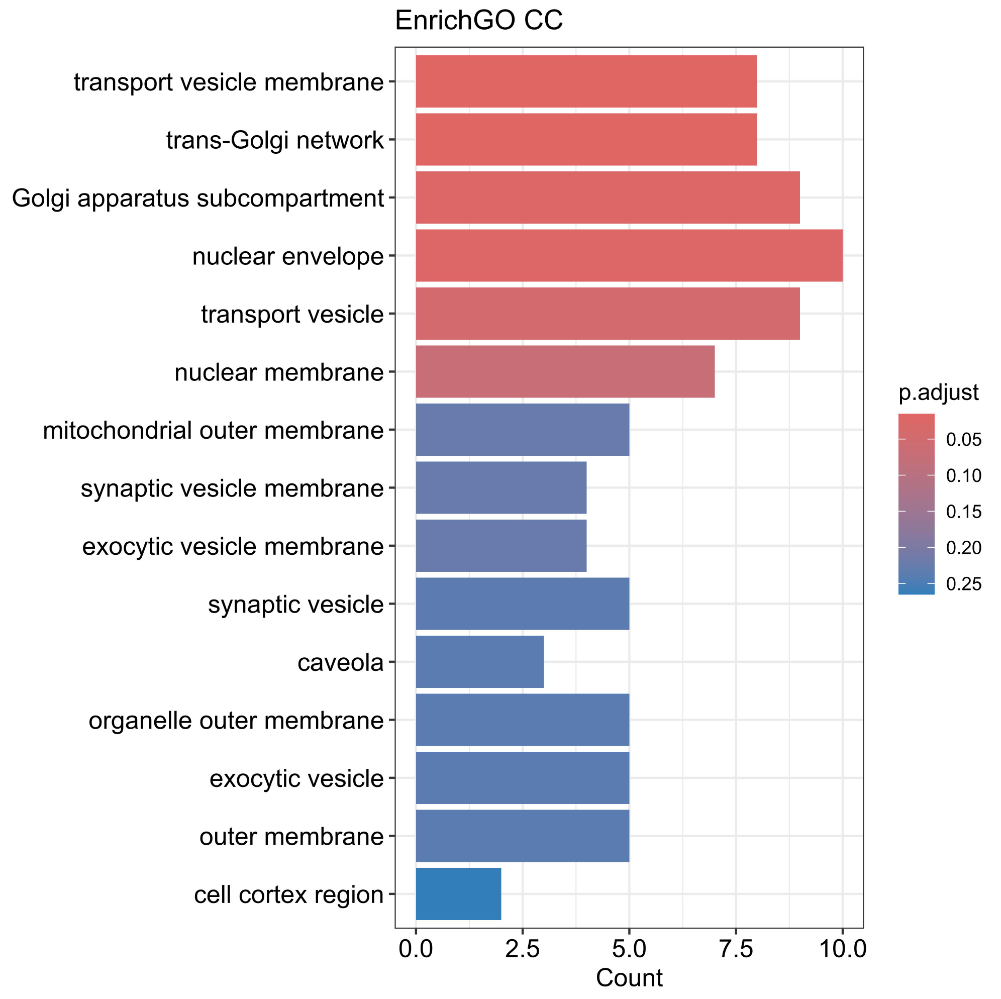
**

**Figure S7. The enrichment analysis results of NSCLC-specific sex-biased RI related genes**

| **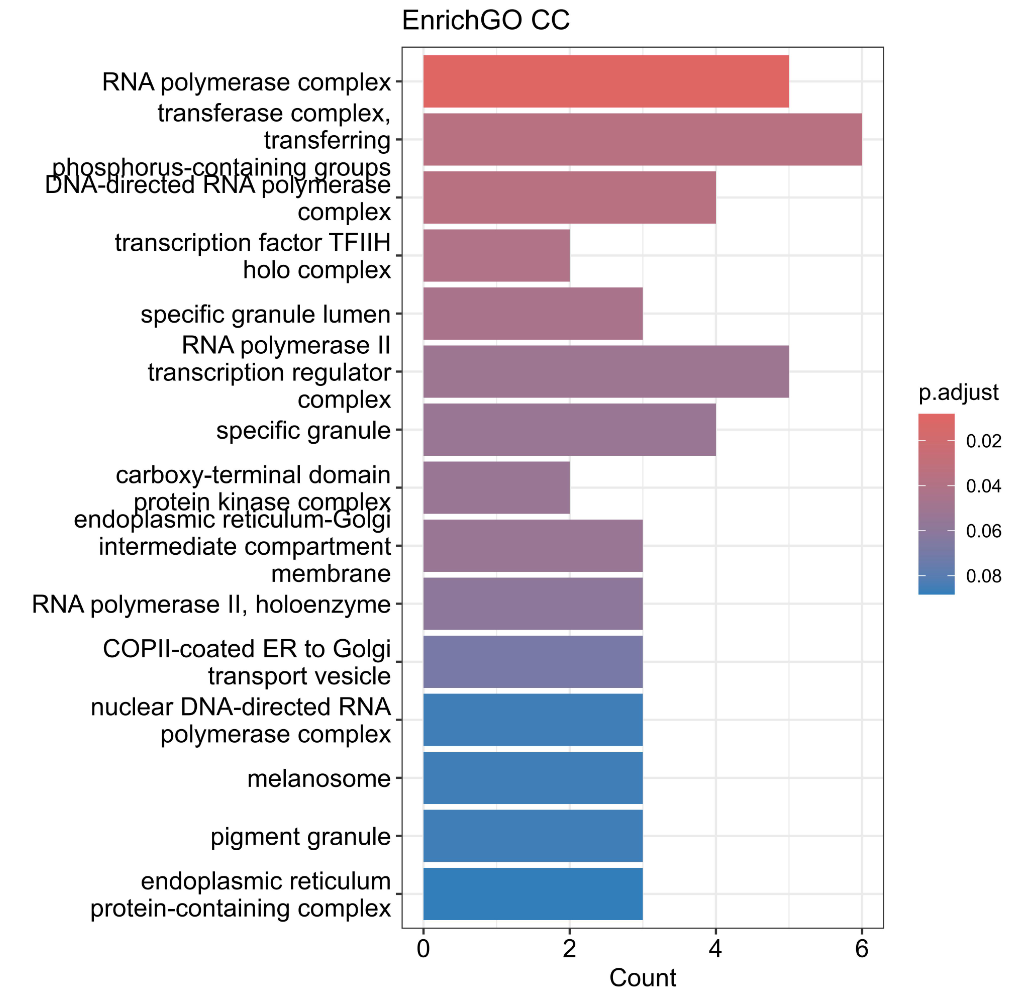** | **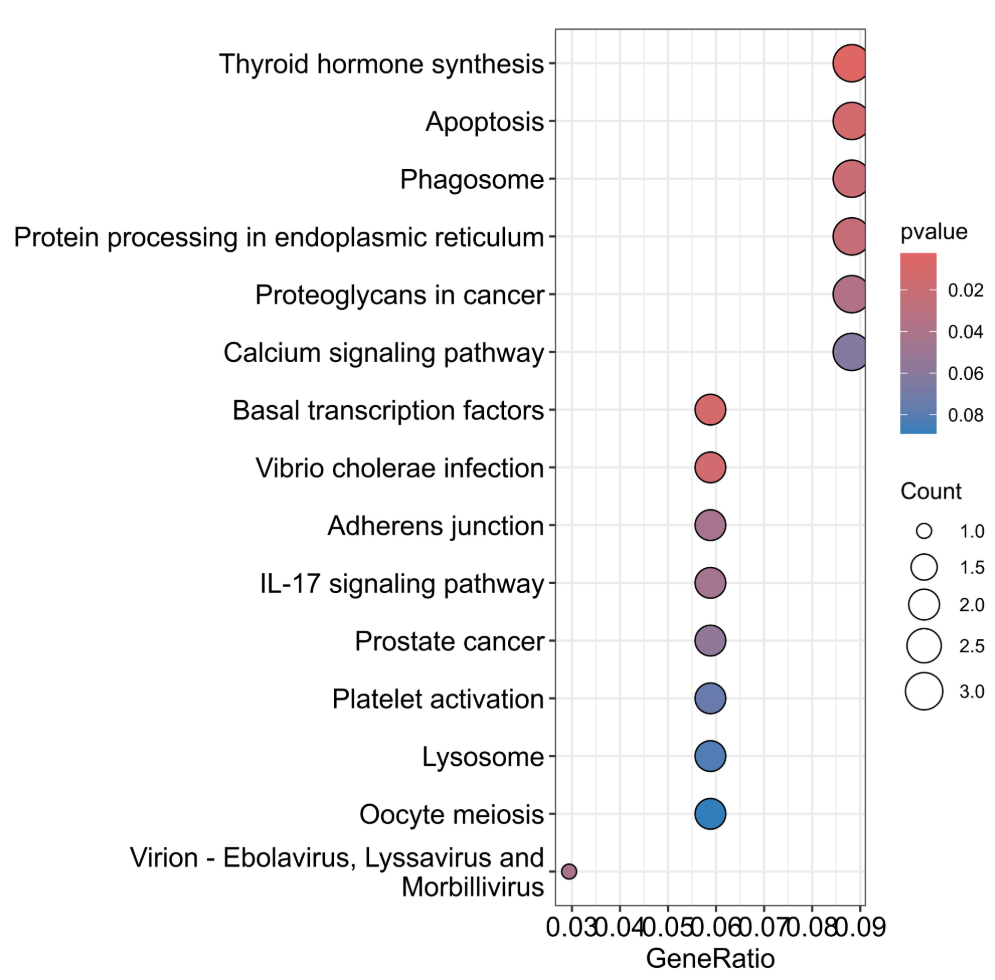** |
| --- | --- |
